# Geospatial disparities and predictors of anaemia among pregnant women in Sub-Saharan Africa

**DOI:** 10.1186/s12884-023-06008-3

**Published:** 2023-10-20

**Authors:** Samuel H. Nyarko, Ebenezer N.K Boateng, Kwamena S. Dickson, David Adzrago, Isaac Y. Addo, Evelyn Acquah, Castro Ayebeng

**Affiliations:** 1 Department of Epidemiology, Human Genetics & Environmental Sciences, School of Public Health, The University of Texas Health Science Center at Houston (UTHealth), Houston, TX USA; 2 Department of Geography and Regional Planning, University of Cape Coast, Cape Coast, Ghana; 3 Department of Population and Health, University of Cape Coast, Cape Coast, Ghana; 4 Center for Health Promotion and Prevention Research (CHPPR), School of Public Health, The University of Texas Health Science Center at Houston (UTHealth), Houston, TX USA; 5 Centre for Social Research in Health, The University of New South Wales, Sydney, Australia; 6 Centre for Health Policy and Implementation Research, Institute of Health Research, University of Health, and Allied Sciences, Ho, Ghana

**Keywords:** Geospatial, Anaemia, Pregnant women, Sub-Saharan Africa

## Abstract

**Background:**

Anaemia has become a major public health concern among women in Sub-Saharan Africa (SSA). However, little is known about the spatial disparities in anaemia prevalence and their associated factors among pregnant women in the region. This study analysed the spatial disparities in anaemia and their associated factors among pregnant women in rural and urban settings in SSA.

**Methods:**

This is a secondary analysis of the most recent demographic and health surveys of 26 countries in SSA. Spatial autocorrelation and hotspot assessment were conducted, while a multivariate logistic regression model was used to identify demographic factors associated with anaemia.

**Results:**

Anaemia was reported among ~50% of pregnant women in urban and rural areas of SSA. The hotspot analysis identified the West African sub-region as having a higher concentration of anaemia cases in rural settings. In urban areas, the odds of anaemia were significantly higher among pregnant women in their second trimester (Adjusted OR = 2.39, CI = 1.99, 2.76). On the other hand, pregnant women in their third trimester (Adjusted OR = 1.98, CI = 1.77, 2.22) and those who had taken intestinal parasite drugs (Adjusted OR = 1.12 CI = 1.02, 1.23) had a higher likelihood of having anaemia in rural areas. Pregnant women aged 35–39 years (Adjusted OR = 0.52, CI = 0.33, 0.81) and those aged 40–44 years (Adjusted OR = 0.69, CI = 0.50, 0.95) had a lesser likelihood of having anaemia compared to women aged 15–19 years in urban and rural areas respectively. Compared to Congo DR, Benin (OR = 2.22, CI = 1.51, 3.28) and Mali (OR = 3.71, CI = 2.73, 5.05) had higher odds of anaemia in urban and rural areas respectively.

**Conclusions:**

Spatial disparities in anaemia persist among pregnant women in rural and urban settings in SSA. Prevailing spatial variations in anaemia may be addressed by specialised interventions considering the contextual residential settings and socio-economic factors highlighted in this study.

## Introduction

Anaemia in pregnancy refers to a haematological disorder with a haemoglobin concentration of less than 11 g/dL that affects the normal functioning of the body’s organs by creating a scarcity of oxygen that reaches the various tissues and organs of the body [1]. Anaemia is recognised as one of the commonest haematological illnesses in pregnancy [2, 3], and it is usually caused by iron and folic acid deficiency in the body [2].

Anaemia in pregnancy can lead to enormous health complications for both pregnant women and their children [3]. Evidence shows that pregnant women with anaemia usually experience delivery problems, such as preterm births and low birth weight [4]. Untreated anaemia during pregnancy can also result in adverse post-delivery effects, such as impaired child development, learning difficulties, impaired physical performance, and poor quality of life [4]. In 2019, the global prevalence of anaemia was estimated to be 29.6% among non-pregnant women of reproductive age and 36.5% among pregnant women [4]. Reports by the WHO further indicate that the prevalence of severe anaemia during pregnancy is highest (more than 40%) in Sub-Saharan Africa (SSA) compared with the rest of the world [5]. Thus, addressing the prevalence of severe anaemia during pregnancy in SSA is crucial for reducing global anaemia prevalence and promoting optimal health for pregnant women and their foetuses.

A considerable number of studies have further indicated complex variations in anaemia prevalence and factors associated with the condition among rural and urban dwellers [6–10]. In India, for instance, a study found that anaemia prevalence was higher among adolescent girls in urban areas than in rural areas [9], whereas the reverse was seen in a study of an adult population in Ethiopia [8]. Moreover, a study in Haiti has revealed that factors associated with anaemia vary by urban and rural residency due to differential access to resources, including health services and information [7].

Despite the growing indications of spatial variations in the prevalence of anaemia, limited studies, especially in the context of SSA, have explored the urban and rural disparity in anaemia prevalence among pregnant women. In addition, most previous studies on anaemia among pregnant women in SSA, except for a study by Weze et al. [11], are focused on single countries - such as Uganda [12], Nigeria [13], Ethiopia [14], Ghana [15], and Kenya [16]. The study undertaken by Weze et al. [11], while deserving commendation, has areas that require further exploration. Particularly noteworthy is the study’s limited exploration of the intricate geospatial differentials potentially arising from the dichotomy between urban and rural residency settings. Additionally, the temporal confines of the study, spanning from 2000 to 2018, may have created limitations in terms of the currency of the findings. Therefore, a more refined and narrower temporal range encompassing the period from 2010 to 2019, would be necessary. With specific regard to the regression model adopted for pregnant women in the study [11], the chosen variables for inclusion, encompassing age, pregnancy duration, iron supplementation patterns, antenatal visitation frequencies, educational achievements, and parity status, are laudable. However, a more holistic elucidation of the determinants governing anaemia warrants the incorporation of supplementary social determinant variables that are likely to wield substantial influence over anaemia prevalence dynamics. The absence of key social determinant variables, such as health insurance coverage, marital status of pregnant women, wealth status, exposure to media messages, and their employment status potentially limits the explanatory power of the study and emerges as an area warranting dedicated scholarly inquiry. In this present study, we sought to bridge the knowledge gap by exploring the prevalence of anaemia among pregnant women in rural and urban areas in 26 SSA countries. We also examined the geospatial variations (hot and cold spot countries) and social determinant factors associated with the observed outcomes. We believe that recognising the disparity in anaemia prevalence across various geospatial parameters and examining key social determinant factors associated with the variations in anaemia prevalence will assist maternal health workers, health practitioners, policymakers, governments, and health advocates in SSA in providing more targeted interventions for the prevention of anaemia in pregnancy, especially in the SSA region.

## Methods

### Data source

This cross-sectional study is based on pooled secondary data from various countries. We used recent demographic and health surveys (DHS) of twenty-six SSA countries conducted between 2010 and 2019. The inclusion of the countries was based on the availability of the outcome of interest in each country’s last wave of the DHS. Hence, countries without anaemia data were excluded from the sample. The DHS is a nationally representative survey of women aged 15 to 49 years in developing countries as part of the global DHS series. The samples were selected using a multistage sampling technique where primary sampling units were randomly selected at the first level, and then households were subsequently selected from each primary sampling unit at the second stage. The DHS provides reliable data on maternal health outcomes alongside several other population health outcomes and is therefore favourable for this analysis. The current analysis is focused on pregnant women during the five years preceding the surveys and includes a pool of 11,574 pregnant women from various countries.

### Study variables and measurements

#### Outcome variable

The outcome of interest was anaemia level among pregnant women. The DHS measured anaemia for pregnant women based on haemoglobin levels such as not anaemic, mild (10.0-10.9 g/dl), moderate (7.0-9.9 g/dl), and severe (< 7.0 g/dl) [17]. Anaemia status was then measured as a binary outcome – non-anaemic and anaemic – by collapsing respondents with mild, moderate, and severe anaemia levels into anaemic.

#### Independent variables

 The independent variables were measured at various levels including maternal level factors such as age (15–19, 20–24, 25–29, 30–34, 35–39, 40–49), level of education (No education, Primary, Secondary, Higher), wealth status (Poorest, Poorer, Middle, Richer, Richest), parity (1, 2, 3, 4, 5 or more), marital status (Never in a union, Married, Cohabitation, Widowed, Divorced, Separated), labour force (Not working, Working), health insurance coverage (No, Yes), antenatal care visits (Less than 8, 8 or more), duration of pregnancy (first trimester, second trimester, third trimester), intestinal parasite drug taken (No, Yes), iron tablets/syrups taken (Yes, No), exposure to mass media (No, Yes) and country of nationality. Source of water was classified as improved or unimproved. Improved drinking-water source (piped to dwelling, plot or yard) comprised piped water connection located inside the user’s dwelling, plot or yard and bottled water. Improved drinking-water source also included other sources such as public taps or standpipes, tube wells or boreholes, protected dug wells, protected springs and rainwater collection; unimproved drinking-water source comprised unprotected dug well, unprotected spring, cart with small tank or drum, surface water (e.g., river, dam, lake, pond, stream, canal, or irrigation channel), and others [18]. Community-level variables such as community socio-economic status and literacy level were also considered and measured as low, moderate, and high. More specifically, the community level socio-economic status mainly comprised household asset and income index which is a composite measure of household wealth that was created by combining information on household possessions, such as type of housing, ownership of durable goods, access to services and the total income of a household. Community literacy level was measured by assessing the educational attainment of individuals within a community. In some contexts, individuals were asked to read a specific text or perform a basic mathematical calculation.

### Geospatial analysis

The proportions of anaemia were estimated from the various countries considered for this study at both rural and urban levels. The estimated proportions were merged with the shapefiles of the African countries, using ArcMap 10.5. The merged data (shapefile and estimated proportions of anaemia) was projected from WGS 1984 to the sinusoidal projection since spatial statistics require data in the projected format before analysis. Considering the objectives of this study, we tested the distribution of anaemia in both rural and urban areas among SSA countries using the spatial autocorrelation (Global Moran’s I) tool in ArcMap 10.5 [19, 20]. This tool was used to examine whether the distribution of anaemia in both rural and urban areas among the SSA countries was random, clustered, or dispersed. A hotspot analysis (Getis-Ord G) was conducted to identify areas with higher or lower prevalence of anaemia while the Anselin Local Moran’s I cluster and outlier analysis was conducted to explore statistically significant spatial outliers [19, 20].

### Statistical analysis

For the descriptive analysis, the proportion of anaemia was estimated by sociodemographic characteristics, and Pearson’s chi-squared test was used to determine the differences among the proportions. For the multivariate analysis, a logistic regression model was used to estimate adjusted odds ratios and 95% confidence intervals to examine the associations between anaemia and the covariates. The analysis was then stratified by place of residence to explore possible differences in covariates across urban and rural settings. A multicollinearity test with a mean-variance inflation factor (VIF) of 2.86 was observed for the analysis. The analysis was sample weighted to cater for over-sampling and under-sampling concerns in the data. All statistical analysis was performed with Stata (Version 14.0).

## Results

### Prevalence of anaemia

A total of 11, 574, pregnant women were used for the study. Of the 11, 574, 26.3% (3,047) were from urban areas while 73.7% (8,527) were from rural areas. The results showed that 5 in 10 pregnant women in both urban and rural areas of Sub-Saharan Africa (SSA) had anaemia. In urban areas, pregnant women aged 40–44 (62.4%), with no formal education (56.2%) of poorest quintile (58.7%), had five or more children (55.8%), never married (52.1%), working (51.2%), with no health insurance (50.8%), attended less than eight antenatal visits (50.5%), took intestinal parasite drugs (50.3%), took iron tablets (50.5%) and had unimproved source of drinking water (50.5%) had higher prevalence of anaemia. On the other hand, in rural areas, pregnant women aged 15–19 years (54.1%), with no formal education (55.7%), of poorest wealth quintile (51.6%), those who had five or more children (52.6%), married (50.7%), working (50.3%), had no health insurance coverage (50.6%), had eight or more antenatal care visits (56.8%), in their third trimester of pregnancy (55.5%), took intestinal parasite drugs (51.4%), took iron tablets (50.2%), of low community socio-economic status (51.8%), had low community literacy desired level (52.3%), had exposure to mass media (50.3%), and had other sources of drinking water (57.0%) had the highest prevalence of anaemia (see Table 1).

**Table 1:** Background characteristics and prevalence of anaemia

Variable	Urban		Rural	
	Frequency (n = 3,047)	Proportion of Anaemia	Frequency (n = 8,527)	Proportion of Anaemia
**Age (years)**	X^2^ = 8.7,p = 0.191		X^2^ = 3.0, p = 0.688	
15–19	158	55.9	525	54.1
20–24	793	50.7	2,251	49.3
25–29	963	48.3	2,391	48.4
30–34	696	51.0	1,772	51.7
35–39	351	46.8	1,126	51.3
40–44	74	62.4	400	49.2
45–49	12	36.6	62	52.1
**Level of education**	X^2^ = 27.863, p < 0.001		X^2^ = 92.237, p < 0.001	
*No education*	659	56.2	4,115	55.6
*Primary*	846	52.3	3,263	44.7
*Secondary*	1,318	47.9	1,100	46.0
*Higher*	224	35.4	49	35.1
**Wealth index**	X^2^ = 22.859, p < 0.001		X^2^ = 13.404, p < 0.001	
*Poorest*	182	58.7	2,620	51.6
*Poorer*	244	54.1	2,493	50.4
*Middle*	443	56.1	1,883	48.6
*Richer*	962	50.9	1,211	50.6
*Richest*	1,216	44.9	320	41.2
**Parity**	X^2^ = 15.391, p < 0.001		X^2^ = 6.939, p = 0.139	
One	958	49.0	1,856	50.3
Two	761	46.5	1,633	47.5
Three	501	48.1	1,437	48.2
Four	328	55.2	1,122	49.9
Five or more	499	55.8	2,509	52.6
**Marital status**	X^2^ = 2.635, p = 0.756		X^2^ = 8.649, p = 0.124	
*Never in union*	118	52.1	132	45.9
*Married*	2,147	49.9	6,751	50.7
*Cohabitation*	679	50.3	1,388	47.7
*Widowed*	9	51.1	40	45.8
*Divorced*	24	50.9	73	44.8
*Separated*	70	46.7	143	48.9
**Labour force**	X^2^ = 0.293, p = 0.588		X^2^ = 0.179, p = 0.672	
*Not working*	1,055	47.7	2,676	49.5
*Working*	1,992	51.2	5,851	50.3
**Health insurance coverage**	X^2^ = 5.263, p = 0.022		X^2^ = 7.840, p = 0.005	
*No*	2,674	50.8	8,020	50.6
*Yes*	373	44.0	507	42.3
**Antenatal care visits**	X^2^ = 1.350, p = 0.245		X^2^ = 0.208, p = 0.648	
*Less than 8*	2,807	50.5	8,406	50.0
*8 or more*	240	44.6	121	56.8
**Duration of pregnancy**	X^2^ = 100.594, p < 0.001		X^2^ = 173.492, p < 0.001	
*1st trimester*	876	35.1	2,243	37.6
*2nd trimester*	1,152	58.5	3,466	53.7
*3rd trimester*	1,019	53.3	2,818	55.5
**Intestinal parasite drugs taken**	X^2^ = 0.065, p = 0.798		X^2^ = 0.484, p = 0.487	
*No*	1,508	49.7	5,094	49.2
*Yes*	1,539	50.3	3,433	51.4
**Iron tablets/syrup taken**	X^2^ = 1.048, p = 0.306		X^2^ = 0.279, p = 0.598	
*No*	472	47.1	2,339	49.8
*Yes*	2,575	50.5	6,188	50.2
**Community socio economic status**	X^2^ = 13.432, p < 0.001		X^2^ = 23.435, p < 0.001	
*Low*	417	57.1	6,240	51.8
*Moderate*	599	57.0	1,840	44.7
*High*	2,031	46.5	437	47.2
**Community literacy level**	X^2^ = 10.895, p = 0.004		X^2^ = 25.512, p < 0.001	
*Low*	373	54.6	4,676	52.3
*Moderate*	959	50.4	3,065	47.9
*High*	1,715	48.8	786	45.6
**Exposure to mass media**	X^2^ = 2.668, p = 0.102		X^2^ = 0.726, p = 0.394	
*No*	726	53.6	3,809	49.7
*Yes*	2,321	48.9	4,718	50.3
**Source of drinking water**	X^2^ = 0.404, p = 0.817		X^2^ = 2.611, p = 0.271	
*Improved*	2,513	50.0	4,560	49.7
*Unimproved*	461	50.5	3,790	50.1
*Other/not in the house*	73	45.6	177	57.0
**Total**	**3,047**	**50.0**	**8,527**	**50.0**

The prevalence of anaemia in pregnant women in urban areas ranged from two in ten pregnant women in Rwanda to seven in ten pregnant women in Mali, while the prevalence among women in rural areas ranged from two in ten to six in ten in Rwanda and Benin respectively (see Table 2).

**Table 2:** Prevalence of anaemia among pregnant women in SSA countries

Country	Urban	95% confidence interval	Rural	95% confidence interval
Burkina Faso	57.2	46.9–67.0	56.9	52.8–61.0
Benin	69.2	62.5–75.2	67.7	62.7–72.4
Burundi	35.5	23.3–50.0	47.2	42.8–51.6
Congo DR	51.3	45.0-57.5	40.9	37.0-44.8
Congo	62.1	53.8–69.8	60.6	52.2–68.4
Cote d’Ivoire	57.0	46.4–67.0	62.7	55.8–69.1
Cameroon	34.7	27.8–42.2	41.4	35.4–47.7
Ethiopia	30.4	19.2–44.5	31.2	27.9–34.7
Gabon	54.2	47.8–60.6	61.3	47.2–73.7
Ghana	37.7	28.4–48.4	57.0	47.7–65.8
Gambia	48.1	40.9–55.3	67.5	58.0-75.8
Guinea	52.9	42.4–63.1	44.0	37.5–50.7
Liberia	36.6	26.1–48.5	49.1	38.1–60.1
Mali	60.4	49.1–70.7	72.3	67.2–77.0
Malawi	56.6	39.0-72.9	43.7	38.3–49.2
Mozambique	53.1	46.5–59.6	51.0	47.4–54.5
Nigeria	56.6	51.2–61.9	61.2	56.7–65.4
Niger	58.4	45.6–70.2	61.0	56.7–65.1
Namibia	23.1	13.4–36.7	27.8	16.0-43.7
Rwanda	21.8	12.5–35.3	22.4	16.9–29.0
Sierra Leone	46.0	33.1–59.4	61.5	53.9–68.6
Togo	60.3	50.0-69.8	62.9	55.8–69.4
Tanzania	48.7	41.2–56.3	61.0	55.8–69.4
Uganda	31.7	22.5–42.5	35.7	30.8–41.0
Zambia	43.9	37.3–50.7	38.5	34.2–43.0
Zimbabwe	37.2	27.8–47.7	27.1	21.5–33.6
Total	50.0	48.3–51.7	50.0	48.9–51.0

### Spatial disparities in anaemia prevalence

**Fig. 1:**
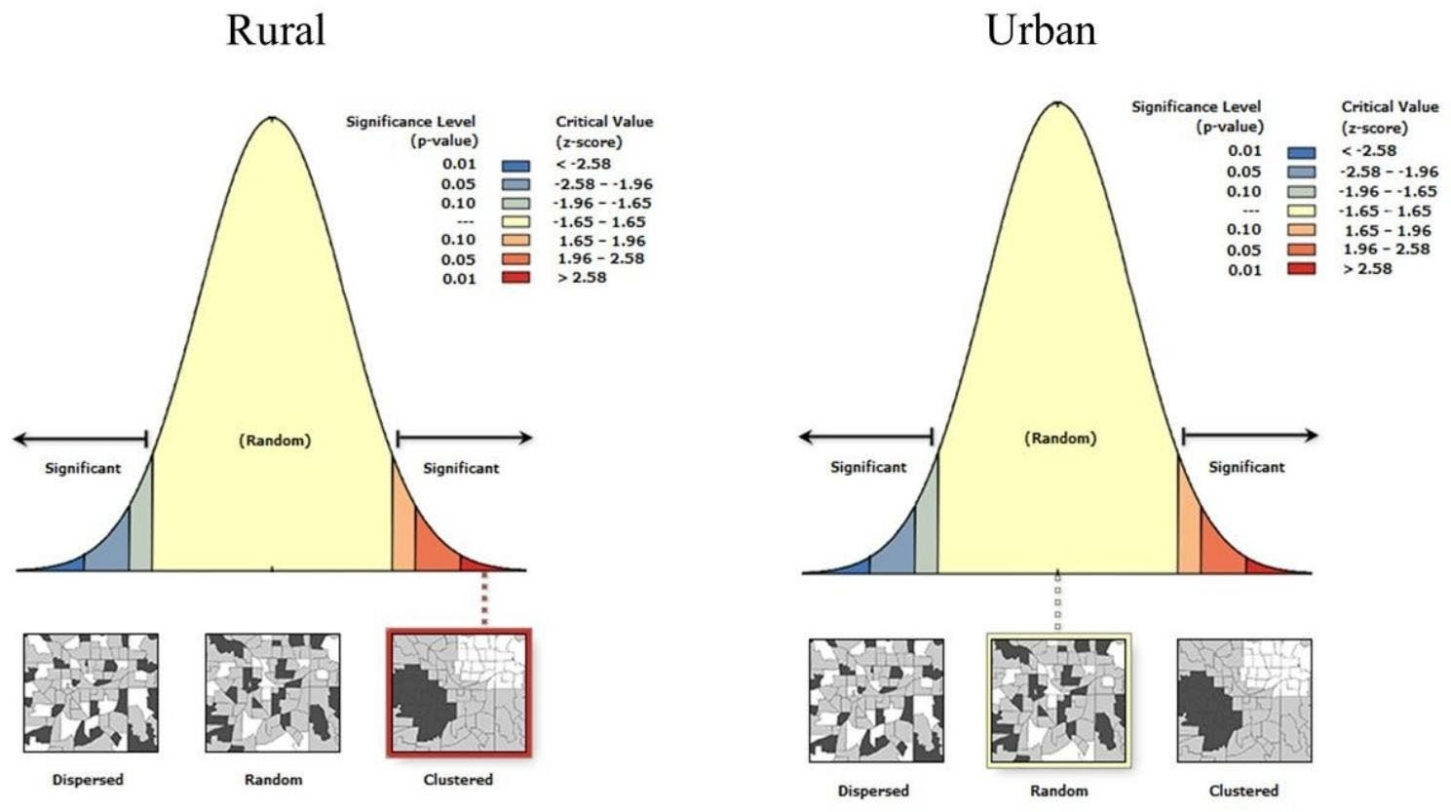
Spatial distribution of anaemia among selected Sub-Saharan African countries

Results from the spatial autocorrelation revealed that the distribution of anaemia in both urban and rural areas among the included SSA countries was random and clustered, respectively (Fig. 1). Narrowing down to the clustered incidence of anaemia in rural areas, this is based on Moran’s I index and the z-score or p-value. A positive and negative Moran’s I index value implies the likelihood of clustering and dispersion, respectively. Moran’s I index was 0.336734, and the p-value was 0.001, indicating the clustering of anaemia in rural areas among the selected SSA countries. The null hypothesis was rejected since Moran’s I index showed that there is clustering which indicates the non-random distribution of anaemia in rural areas among the selected SSA countries. The z-score obtained (3.183726) was > 2.58 and indicated statistical significance at a 99% confidence level. The distribution of anaemia among the SSA countries is clustered in rural areas.

**Fig. 2:**
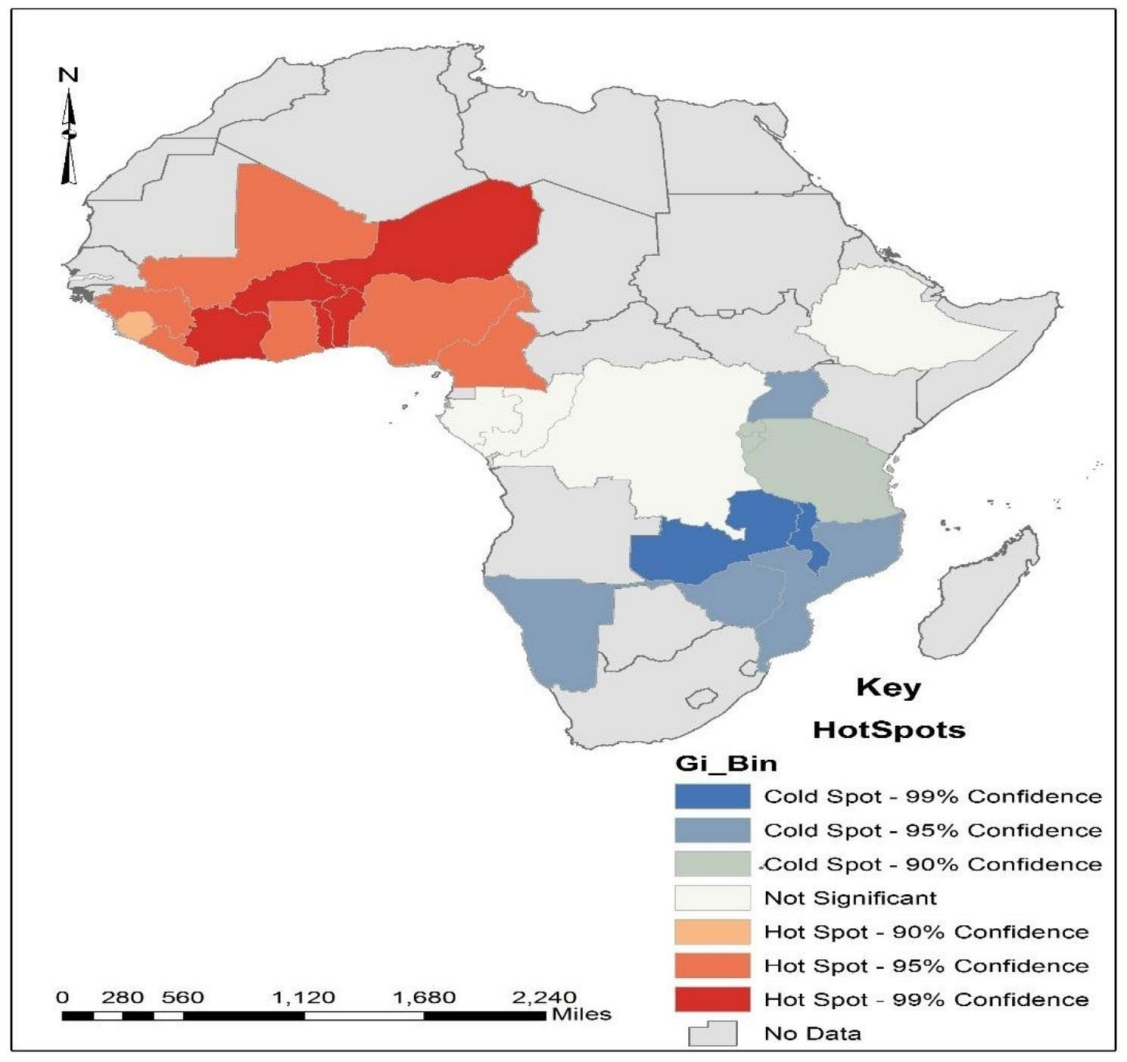
Hotspot analysis of anaemia distribution in sub-Saharan Africa

The Getis-Ord G hotspot analysis revealed that countries in West Africa are hotspot zones for anaemia incidence in SSA (Fig. 2). Countries, such as Cote D’Ivoire, Burkina Faso, Togo, Benin, and Niger were found to be the hotspots for anaemia incidence in rural areas at a 99% confidence level. In addition, at a 95% confidence level, countries such as Ghana, Cameroon, Mali, Guinea, and Liberia were also found to be the hotspots. On the contrary, at a 95% confidence level, countries such as Uganda, Zimbabwe, Mozambique, and Namibia were found to be coldspots for the incidence of anaemia in rural areas. Countries such as Zambia and Malawi were found to be coldspots for the incidence of anaemia in rural areas at a 99% confidence level.

**Fig. 3:**
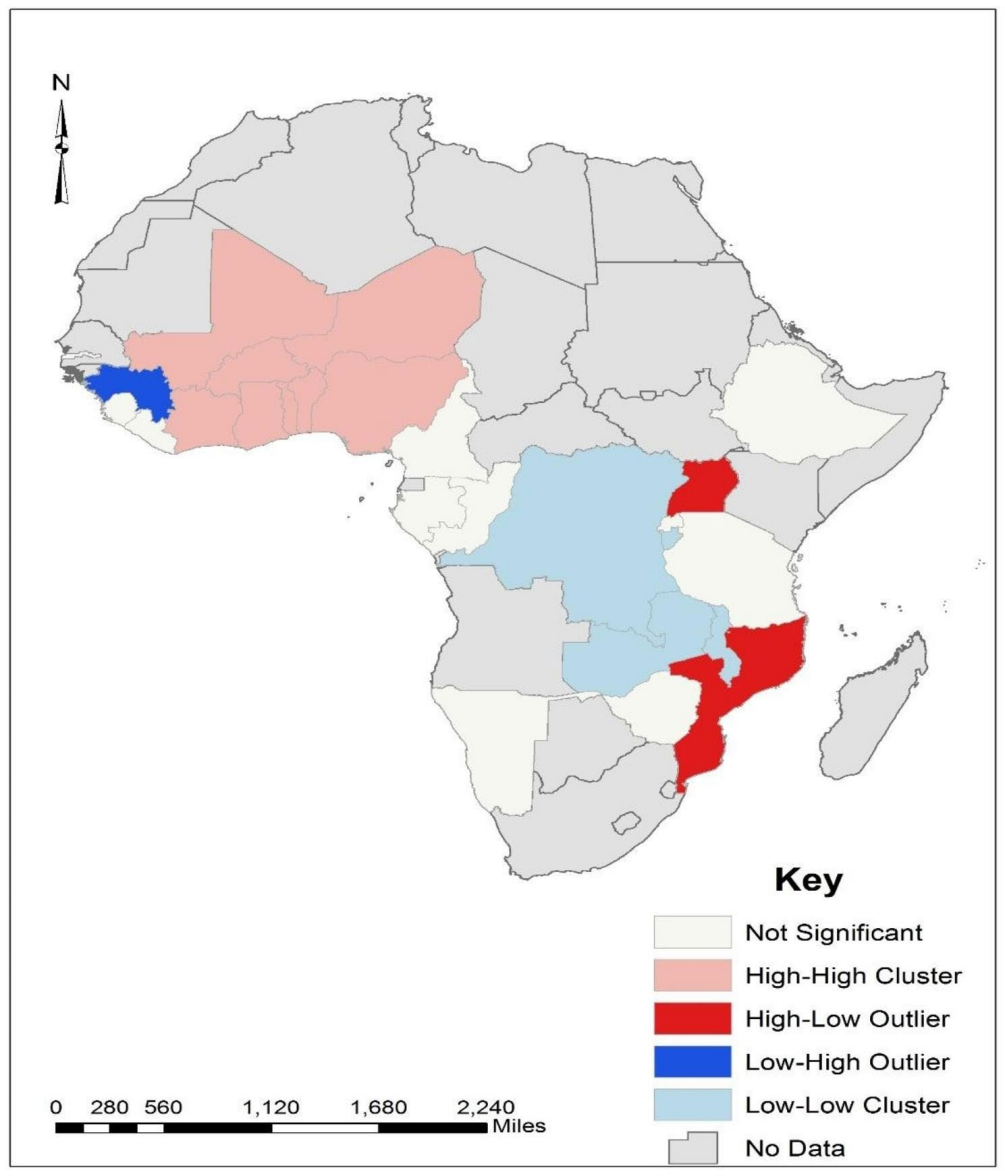
Cluster and outlier analysis of anaemia distribution in Rural Africa

The cluster and outlier analysis (Fig. 3) showed that countries such as Ghana, Cote D’Ivoire, Togo, Benin, Nigeria, Niger, Mali, and Burkina Faso had a high incidence of anaemia and were surrounded by countries with a high incidence of anaemia in their rural areas. This validates the finding from the hotspot analysis (Fig. 2) and implies that there is a high incidence of anaemia in the rural areas of such countries. It was also found that countries such as Uganda and Mozambique had a high incidence of anaemia in their rural areas but were surrounded by countries with a lower incidence of anaemia in their rural areas. DR Congo and Zambia were found to have a low incidence of anaemia in their rural areas and are surrounded by countries with a low incidence of anaemia in their rural areas. Furthermore, Guinea was found to be the only country with a low incidence of anaemia in its rural areas but surrounded by countries with a high incidence of anaemia in their rural areas. The purpose of this analysis was to examine the uncaptured information of outliers which is not identified by the hotspot analysis. Therefore, although most countries in the West African sub-region had a high incidence in their rural areas, a country such as Guinea has a low incidence of anaemia (see Fig. 3).

### Multivariable analysis of anaemia among pregnant women in SSA

The logistic regression of anaemia among pregnant women in SSA is presented in Table 3. The results showed significant associations between anaemia in pregnancy and age, level of education, and duration of pregnancy in urban areas, while a significant association between anaemia in pregnancy and age, level of education, duration of pregnancy, intestinal parasite drug intake, community socioeconomic status and community literacy level was observed in rural areas. The logistic regression model revealed that pregnant women aged 35–39 years were less likely to have anaemia in urban areas (AOR = 0.52, CI = 0.33, 0.81) compared to those aged 15–19 years. Pregnant women with a higher level of formal education (AOR = 0.58, CI = 0.41, 0.84) were less likely to have anaemia in urban areas compared to those with no formal education. The likelihood of pregnant women in the second trimester having anaemia was high (AOR = 2.39, CI = 1.99, 2.76) compared to those in the first trimester in urban areas. Pregnant women aged 40–44 years had a lesser (AOR = 0.69, CI = 0.50, 0.95) likelihood of anaemia in rural areas compared to those aged 15–19 years. The likelihood of pregnant women with a higher level of formal education having anaemia was less (AOR = 0.62, CI = 0.35, 1.11) compared to those with no formal education in rural areas. Pregnant women in the third trimester were more (AOR = 1.98, CI = 1.77, 2.22) likely to have anaemia compared to those in the first trimester in rural areas. The likelihood of pregnant women taking intestinal parasite drugs and having anaemia was high (AOR = 1.12, CI = 1.02, 1.23) compared to those not taking intestinal parasite drugs in rural areas. Pregnant women with moderate community socio-economic status had a lesser (AOR = 0.83, CI = 0.73, 0.93) likelihood of having anaemia compared to those with low community socio-economic status in rural areas. The likelihood of pregnant women with moderate community literacy levels having anaemia was less (AOR = 0.90, CI = 0.82, 0.99) compared to those with low community literacy levels in rural areas (see Table 3).

**Table 3:** Logistic regression analysis of anaemia among pregnant women in SSA

Variable	Urban	Rural
	Adjusted Odds Ratio (95% Confidence interval)	Adjusted Odds Ratio (95% Confidence interval)
**Age (years)**		
15–19	Ref	Ref
20–24	0.77 (0.54, 1.11)	0.88 (0.72, 1.08)
25–29	0.65**(0.44, 0.95)	0.78**(0.62, 0.97)
30–34	0.69(0.46, 1.04)	0.78*(0.61, 0.99)
35–39	0.52**(0.33, 0.81)	0.77 (0.59, 1.01)
40–44	0.69(0.37, 1.29)	0.69**(0.50, 0.95)
45–49	0.78(0.22, 2.77)	0.79(0.45, 1.39)
**Level of education**		
*No education*	Ref	Ref
*Primary*	0.81*(0.66, 0.99)	0.67***(0.60, 0.74)
*Secondary*	0.74***(0.60, 0.91)	0.76***(0.65, 0.87)
*Higher*	0.58**(0.41, 0.84)	0.62(0.35, 1.11)
**Wealth index**		
Poorest	Ref	Ref
Poorer	0.96(0.66, 1.40)	0.92(0.83, 1.03)
Middle	0.97(0.69, 1.38)	0.92(0.81, 1.04)
Richer	0.86(0.61, 1.22)	1.00(0.85, 1.16)
Richest	0.79(0.55, 1.13)	0.84(0.64, 1.12)
**Parity**		
*One*	Ref	Ref
*Two*	0.92 (0.75, 1.14)	0.96 (0.83, 1.11)
*Three*	1.05 (0.81, 1.35)	0.96 (0.82, 1.14)
*Four*	1.24 (0.92, 1.66)	1.05 (0.87, 1.27)
*Five or more*	1.33 (0.98, 1.80)	1.09 (0.90, 1.32)
**Marital status**		
*Never in union*	Ref	Ref
*Married*	0.86 (0.58, 1.29)	1.29 (0.94, 1.77)
*Cohabitation*	0.92 (0.61, 1.40)	1.32 (0.95, 1.83)
*Widowed*	1.38 (0.41, 4.70)	0.97 (0.46, 2.06)
*Divorced*	0.51 (0.20, 1.34)	0.99 (0.56, 1.74)
*Separated*	0.81 (0.44, 1.47)	1.25 (0.81, 1.94)
**Labour force**		
*Not working*	Ref	Ref
*Working*	1.08(0.92, 1.27)	1.05 (0.96, 1.16)
**Health insurance coverage**		
*No*	Ref	Ref
*Yes*	0.89 (0.70, 1.12)	0.87 (0.72, 1.04)
**Antenatal care visits**		
*Less than 8*	Ref	Ref
*8 or more*	1.07 (0.79, 1.47)	1.07 (0.76, 1.53)
**Duration of pregnancy**		
*1st trimester*	Ref	Ref
*2nd trimester*	2.39***(1.99, 2.88)	1.81***(1.63, 2.02)
*3rd trimester*	2.28***(1.89, 2.76)	1.98***(1.77, 2.22)
**Intestinal parasite drug taken**		
*No*	Ref	Ref
*Yes*	1.00 (0.85, 1.16)	1.12**(1.02, 1.23)
**Iron tablets/syrup taken**		
*No*	Ref	Ref
*Yes*	1.12 (0.91, 1.38)	0.99 (0.89, 1.10)
**Community socioeconomic status**		
*Low*	Ref	Ref
*Moderate*	0.94 (0.72, 1.23)	0.83**(0.73, 0.93)
*High*	0.89 (0.68, 1.18)	0.97 (0.77, 1.22)
**Community literacy level**		
*Low*	Ref	Ref
*Moderate*	1.21(0.94, 1.56)	0.90*(0.82, 0.99)
*High*	1.11 (0.85, 1.45)	0.97 (0.77, 1.22)
**Exposure to mass media**		
*No*	Ref	Ref
*Yes*	1.03 (0.86, 1.24)	1.04 (0.95, 1.14)
**Source of drinking water**		
*Improved*	Ref	Ref
*Unimproved*	0.96 (0.78, 1.20)	0.95(0.87, 1.04)
*Other/not in the house*	0.90 (0.57, 1.44)	1.30(0.97, 1.74)

Ref = Reference category *p < 0.05 **p < 0.01 ***p < 0.001

### Logistic regression analysis of anaemia among pregnant women by countries in SSA

The result showed that among urban areas, the likelihood of anaemia among pregnant women was high in Benin (OR = 2.22, CI = 1.51, 3.28) compared to Congo DR. On the other hand, the likelihood of anaemia among pregnant women in rural areas was high in Mali (OR = 3.71, CI = 2.73, 5.05) compared to Congo DR. Pregnant women in Rwanda were less likely to have anaemia in both rural (OR = 0.35, CI = 0.24,0.53) and urban areas (OR = 0.25 CI = 0.12, 0.53) (see Table 4).

**Table 4:** Logistic regression analysis of anaemia among pregnant women by countries in SSA

Country	Urban	Rural
	Odds Ratio (95% Confidence Level)	Odds Ratio (95% Confidence Level)
Burkina Faso	1.26(0.79, 2.00)	1.59***(1.26, 2.01)
Benin	2.22***(1.51, 3.28)	2.64***(2.01, 3.46)
Burundi	0.58*(0.34, 0.99)	1.92***(1.46, 2.53)
Congo DR	Ref	Ref
Congo	1.61 (0.94, 2.77)	1.92***(1.46, 2.53)
Cote d’Ivoire	1.10 (0.68, 1.80)	1.93***(1.40, 2.65)
Cameroon	0.53**(0.35, 0.81)	0.94 (0.68, 1.26)
Ethiopia	0.55**(0.34, 0.90)	0.89(0.72, 1.11)
Gabon	1.37 (0.94, 2.00)	2.05***(1.41, 2.96)
Ghana	0.71 (0.43, 1.17)	1.97***(1.34, 2.88)
Gambia	0.85 (0.56, 1.29)	2.79***(1.98, 3.93)
Guinea	1.10 (0.68, 1.79)	1.10(0.80, 1.50)
Liberia	0.88 (0.45, 1.70)	1.41(0.98, 2.03)
Mali	1.53 (0.93, 2.53)	3.71***(2.73, 5.05)
Malawi	0.83 (0.42, 1.63)	0.97(0.74, 1.28)
Mozambique	1.01 (0.70, 1.46)	1.38**(1.11, 1.71)
Nigeria	1.39 (0.98, 1.97)	2.44***(1.92, 3.10)
Niger	1.28 (0.79, 2.08)	1.94***(1.52, 2.47)
Namibia	0.25***(0.12, 0.52)	0.44**(0.22, 0.88)
Rwanda	0.25***(0.12, 0.53)	0.35***(0.24, 0.53)
Sierra Leone	0.93 (0.51, 1.70)	2.11***(1.49, 2.97)
Togo	1.02 (0.68, 1.53)	1.90***(1.38, 2.60)
Tanzania	1.02 (0.68, 1.53)	2.06***(1.64, 2.59)
Uganda	0.41**(0.23, 0.73)	0.76*(0.59, 0.99)
Zambia	0.45***(0.29, 0.68)	0.75**(0.59, 0.95)
Zimbabwe	0.40**(0.24, 0.67	0.48***(0.33, 0.70)

Ref = Reference category *p < 0.05 **p < 0.01 ***p < 0.001

## Discussion

Using multivariate modelling and geospatial analysis, this study examined the prevalence of anaemia among pregnant women in rural and urban areas in 26 Sub-Saharan Africa (SSA) countries. Our analysis shows that 5 in 10 pregnant women in both urban and rural areas of SSA had anaemia. The prevalence of anaemia in urban areas ranged from two in ten to seven in ten, while the prevalence in rural areas ranged from two in ten to six in ten. The present findings imply that holistically the risks of developing anaemia are similar between urban and rural dwellers in SSA. However, the risk levels vary significantly among the various individual and community-level characteristics of pregnant women. Specifically, we found that age, level of education, duration of pregnancy, intestinal parasite drug intake, community socioeconomic status, and community literacy level explained the variations in anaemia across urban and rural areas in SSA.

Consistent with previous studies [1, 21], older age at pregnancy (20 years and over) was associated with lower odds of anaemia, whereas those younger (15–19 years) were more at risk of developing anaemia in urban and rural areas across SSA. The evidence further suggests that pregnant adolescents often have lower haemoglobin levels that lead to anaemia due to increased micronutrient demands and lack of nutrition uptake or worsening irregular eating behaviour [11, 22]. The high burden of anaemia among these younger pregnant women might be due to a lack of socioeconomic opportunities (e.g., education, employment, etc.) to afford healthy lifestyles (healthy eating, health resources), which reduce the risk of anaemia. This health disparity is concerning because adolescent pregnancy is high in SSA, predisposing these young women to anaemia and other risk factors such as preterm births, low birth weight, and obstetric complications [11, 22, 23]. Providing dietary intake or maternal nutrition education while educating young women about the complications of adolescent pregnancy may help reduce and prevent anaemia in SSA, especially among young women.

Gestational age or duration of pregnancy was identified as another critical factor associated with the risk of developing anaemia among women in rural and urban areas in SSA. Pregnant women in their second and third trimesters were more likely to develop anaemia compared to those in their first trimester. This finding is similar to reported outcomes in some previously published studies [24–28]. This increased risk could be due to poor adherence to iron supplementation and antenatal care (ANC) service utilisation among pregnant women, which are common problems in SSA [11].

The educational status of communities and pregnant women is necessary for improving economic stability, nutritional requirement during pregnancy, antenatal care (ANC) service utilisation, and averting anaemia [29–31]. We found in our study that pregnant women in rural areas with moderate literacy levels or socioeconomic status were less likely to experience anaemia than those with low literacy levels or socioeconomic status. Women in SSA communities with high illiteracy levels may be at a higher risk of anaemia than in communities with high literacy levels [1, 32, 33]. The limited educational and women empowerment opportunities in some SSA communities might have decreased pregnant women, an underserved population, access to health information and resources that improve awareness about and treatment of health conditions (e.g., anaemia). Our findings consistently demonstrate that pregnant women in both rural and urban areas with at least primary education were less likely to develop anaemia compared to their counterparts with no education.

Anaemia often occurs due to increased risks of infections (e.g., intestinal parasites, diarrhoea, gut inflammation from parasitic infestations, and subclinical microbial infections), especially in developing countries, including SSA [11, 34, 35]. Although antiparasitic infection medications are used to treat some intestinal parasites [36], our findings revealed that pregnant women in rural areas who had taken intestinal parasite drugs were more likely to develop anaemia. These pregnant women might not be taking the recommended doses of the intestinal parasite drugs, thereby limiting the effectiveness of the drugs in treating the infections and potentially preventing anaemia [36, 37]. Further studies are needed to examine the associations between doses of intestinal parasite drugs taken and the risk of developing anaemia in this sample.

Our geospatial analysis demonstrated that West African countries, such as Ghana, Burkina Faso, Mali, Cote D’Ivoire, Togo, Benin, and Niger were hotspots for anaemia incidence among pregnant women in rural areas. The Anselin Local Moran’s I cluster and outlier analysis revealed that although the incidence of anaemia among pregnant women in rural areas in Ghana was low, her surrounding countries such as Mali, Burkina Faso, Togo, Benin, Niger, and Nigeria had a high incidence of anaemia. The cold spot areas for anaemia in rural areas were found among Southern African countries such as Uganda, Zimbabwe, and Namibia. The Anselin Local Moran’s I cluster and outlier analysis further indicated that DR Congo and Uganda had a low incidence of anaemia and were also surrounded by countries with a low incidence of anaemia during pregnancy in rural areas. The higher burdens of anaemia in West African countries compared to the Southern African countries might be due to the increased burdens of malaria and schistosomiasis, which often co-occur with anaemia [38, 39]. However, further studies on anaemia among pregnant women in rural areas in specific countries in SSA may help understand and provide detailed information on the dynamics of anaemia among pregnant women and their risk factors in each of those countries.

Despite the novel contribution of our study as the first to examine anaemia in pregnancy across rural and urban areas in SSA using multivariate and geospatial analytical techniques, the following limitations should be considered when interpreting the data. Anaemia and the characteristics of the study participants were self-reported and may be subject to reporting bias, leading to potential underestimation or overestimation of the findings. The study employed collapsed anaemia as the outcome, which has marked knowledge of the severity of anaemia among the respondents. Also, due to irregularities in the reporting years for DHS data and the limited data on anaemia and some independent variables for some SSA countries, we could include only 26 countries. Hence, the generalisability of our findings is limited. We also analysed secondary cross-sectional data, hence we could not claim any causality in this study.

### Implications for policy and research

The study findings underscore the importance of targeted public health interventions for maternal nutrition education and support to improve haemoglobin levels to address the high anaemia in SSA. This should address issues like poor nutrition intake and irregular eating habits that contribute to anaemia risk. Additionally, specific strategies are needed to ensure proper adherence to iron supplementation and antenatal care (ANC) services, especially among women in their second and third trimesters. Strengthening ANC services, promoting iron-rich diets, and educating women about their significance can help decrease anaemia prevalence. Furthermore, policies should underscore community-level education and empowerment to enhance literacy rates and socioeconomic status, especially in rural areas. Educated women face a lower risk of anaemia, making education promotion for pregnant women and communities vital in reducing anaemia rates. Ensuring accurate dosages and effectiveness of antiparasitic medications is crucial, particularly in rural areas. Monitoring and facilitating effective medication usage can mitigate anaemia risks linked to parasitic infections.

Given the geographic distribution of anaemia, policies should target interventions in identified hotspots like West African countries (Cote D’Ivoire, Burkina Faso, Togo, Benin, and Niger at 99% confidence level; and Ghana, Cameroon, Mali, Guinea, and Liberia at 95% confidence level) with high anaemia rates. These interventions could encompass tailored healthcare services, improved nutrition access, and managing co-occurring conditions like malaria and schistosomiasis. Encouraging further research to grasp anaemia trends in specific countries is also important. This research will aid in crafting interventions that suit local factors influencing anaemia prevalence, leading to more impactful strategies.

## Conclusions

Based on the findings, 50% of pregnant women in SSA experience anaemia, irrespective of rural or urban residence. Anaemia prevalence varies across several socio-demographic characteristics, with age 25–29 years, primary and secondary education levels, trimester pregnancy stages, rural dwellers who had received intestinal parasite drugs, moderate socioeconomic status, and literacy level in rural areas significantly associated with anaemia. The geospatial analysis identifies that the distribution of anaemia among pregnant women in rural areas in SSA is clustered with a few countries in West Africa (Cote D’Ivoire, Burkina Faso, Togo, Benin, and Niger at 99% confidence level; Ghana, Cameroon, Mali, Guinea, and Liberia at 95% confidence level) labelled as hot spots for anaemia in pregnancy. On the contrary, countries such as Uganda, Zimbabwe, Mozambique, Namibia, Zambia and Malawi were found to be cold spots for incidence of anaemia in rural areas, while others like Uganda show low prevalence. Tailored policies are needed for high-prevalence regions to mitigate disparities. While these findings are useful for planning and developing interventions against anaemia in pregnancy, further qualitative exploration is recommended for deeper contextual understanding.

## Data Availability

Data for the current study are accessible at the DHS data repository: https://dhsprogram.com/data/available-datasets.cfm.

## Abbreviations

ANC: Antenatal Care
AOR: Adjusted Odds Ratios
DHS: Demographic and Health Survey
SSA: Sub-Saharan Africa
VIF: Variance Inflation Factor
WGS: World Geodetic System
WHO: World Health Organization
